# Carbon Dioxide (CO_2_) Dietary Emissions Are Related to Oxidative and Inflammatory Status in Adult Population

**DOI:** 10.3390/nu15245050

**Published:** 2023-12-08

**Authors:** Margalida Monserrat-Mesquida, Cristina Bouzas, Silvia García, Maria Magdalena Quetglas-Llabrés, David Mateos, Lucía Ugarriza, Cristina Gómez, Antoni Sureda, Josep A. Tur

**Affiliations:** 1 Research Group on Community Nutrition & Oxidative Stress, University of the Balearic Islands-IUNICS, 07122 Palma de Mallorca, Spain; margalida.monserrat@uib.es (M.M.-M.); silvia.garcia@uib.es (S.G.);; 2 CIBEROBN (Physiopathology of Obesity and Nutrition), Instituto de Salud Carlos III, 28029 Madrid, Spain; 3 Health Research Institute of Balearic Islands (IdISBa), 07120 Palma de Mallorca, Spain; 4 C.S. Camp Redó, IBSalut, 07010 Palma de Mallorca, Spain; 5 Clinical Analysis Service, University Hospital Son Espases, 07198 Palma de Mallorca, Spain

**Keywords:** greenhouse gas emissions, oxidative stress, inflammation, biomarkers

## Abstract

Background: Carbon dioxide (CO_2_) is a primary greenhouse gas (GHG) causing global temperature to rise. Unsustainable diets induce an increment in the risk of obesity and noncommunicable diseases but also contribute to the global GSG burden. Objective: To assess whether CO_2_ dietary emissions influence the inflammatory and oxidative status of subjects with metabolic syndrome (MetS). Methods: As part of the PREDIMED-Plus study, 100 adults (55–75 years old) from the Balearic Islands, Spain, were recruited and classified according to their dietary CO_2_ emissions. Anthropometric parameters were determined, fasting blood samples were collected and plasma, neutrophils, and peripheral blood mononuclear cells (PBMCs) were obtained. Dietary inflammatory index (DII), adherence to a Mediterranean diet (ADM), fatty liver index (FLI), and estimated glomerular filtration (eGFR) were calculated. Clinical biochemical parameters, blood count, and oxidative stress and inflammatory biomarker levels were also determined. Results: DII was higher in participants with high dietary CO_2_ emissions. Adherence to the MedDiet was inversely associated with CO_2_ emissions. Malondialdehyde (MDA) levels were higher in urine and plasma samples from subjects with high dietary CO_2_ emissions. Reactive oxygen species (ROS) production by PBMCs was greater in participants with high CO_2_ emissions. Interleukin-15, resistin, and leptin plasma levels were increased in participants with high dietary CO_2_ emissions. Conclusion: Dietary CO_2_ emissions influence oxidative status and inflammation in relation to the increased prooxidative and proinflammatory status in PBMCs and plasma. These biomarkers were useful for monitoring diet sustainability and health.

## 1. Introduction

Greenhouse gas (GHG) emissions have risen in the last few decades [1], contributing to climate change and altering the way of life, with carbon dioxide (CO_2_) being the main gas responsible. In this regard, the United Nations 2030 agenda pursues 17 sustainable development goals, among them a reduction in CO_2_ emissions to promote sustainable and equal development [2].

In the last decades, a new lifestyle, characterized as fast paced and stressful, has changed global dietary patterns, affecting how we buy and eat food. This change has brought an increment in the consumption of dense and caloric foods, meat protein, refined cereals and sugars, alcohol, and high-fat processed food, inducing damage to health [3]. These new eating habits have influenced the quantity of CO_2_ in the atmosphere, as emissions from the food system represent around two-thirds of global GHG emissions, seriously impacting environmental degradation [4]. Therefore, there is urgent requirement for a worldwide overhaul of the food system. This is crucial, as a significant portion of the global population suffers from insufficient nourishment, and these unwholesome diets elevate the likelihood of morbidity and mortality. Additionally, they contribute to a higher prevalence of obesity and noncommunicable diseases such as coronary heart disease, stroke, and diabetes [5].

Obesity leads to GHG emissions due to the increased energy needed to sustain excess body weight. Estimates suggest that obesity is associated with a 20% higher level of GHG emissions compared to individuals of normal weight [6]. One strategy to reduce obesity and, in parallel, CO_2_ emissions, could be through the Mediterranean diet (MedDiet), a healthy dietary pattern characterized by a more plant-based diet with limited energy intake and food processing, which can reduce the impact on ecosystems [7]. Obesity is directly associated with metabolic syndrome (SMet) [8], but also with a prooxidative/proinflammatory status [9]. Continued exposure to environmentally significant increases in CO_2_ poses a potential health risk, contributing to inflammation, oxidative stress, endothelial dysfunction, diminished higher-level cognitive abilities, bone demineralization, and kidney calcification [10]. Long-term exposure to elevated levels of CO_2_ could induce weak hypercapnia, hypoxia, and acidosis, which can result in cellular damage via inflammation and/or oxidative stress [11]. Oxidative stress is marked by the elevated generation of reactive oxygen species (ROS), a process that can be regulated by both antioxidant enzymes and nonenzymatic molecules [12]. The inflammatory status is distinguished by elevated levels of proinflammatory cytokines, which are linked to the distribution and quality of adipose tissue throughout the body [12,13]. These inflammatory markers, as well as monocyte chemoattractant protein-1 (MCP-1) and leptin, are deregulated in oxidative stress situations [14,15]. It has been evidenced that high proinflammatory cytokine production and high levels of oxidative stress biomarkers are associated with low adherence to the MedDiet and, consequently, to a low-quality diet [16].

The current study aimed to assess whether CO_2_ dietary emissions influence the oxidative and inflammatory status of subjects with MetS. We wanted to study this issue because it has been seen that prolonged exposure to environmental CO_2_ emissions is related to inflammation and oxidative stress, a potential health risk. So, we examined whether CO_2_ dietary emissions have the same effect as environmental CO_2_ emissions.

## 2. Materials and Methods

### 2.1. Study Design

Within the context of the PREDIMED-Plus trial, 100 individuals aged between 55 and 75 from the Balearic Islands participated. Inclusion criteria involved individuals who were overweight or obese and met at least three of the MetS criteria, as per the updated harmonized definition by the International Diabetes Federation, the American Heart Association, and the National Heart, Lung, and Blood Institute [17]. The study protocol, available elsewhere [18] and on http://predimedplus.com/ (accessed on 6 December 2023), was previously published. The International Standard Randomized Controlled Trial (ISRCT; http://www.isrctn.com/ISRCTN89898870) registered this trial with the number 89898870 in 2014. Trial registration: ISRCTN, ISRCTN89898870. Registered 5 September 2013.

The participants were randomly assigned to two groups based on the CO_2_ emissions generated during the manufacturing and production of food. CO_2_ production was determined by calculating the amount of CO_2_ emitted per kilogram (kg) of food, utilizing a 2016 European database. This database, derived from lifecycle assessments (LCAs) in recent studies, detailed the kilograms of CO_2_ emitted per kilogram of consumed food and encompassed the various stages of production and processing of agricultural products [19]. The calculation of kilograms of CO_2_ emitted per unit of consumed food involved multiplying the grams of each food item reported in the 143-item food frequency questionnaire (FFQ) [20] per kg of CO_2_ emitted per kg of each food from the database. To determine the total daily emission from diet, the sum of all kg of CO_2_ was summed across all foods. After finding out the amount of CO_2_ emitted for each participant, a calculation was made of CO_2_ emitted per 1 kg of food consumed [21]. Therefore, in the present study, the median value was determined by deducting the median kg value of CO_2_ emitted per day, which was 2.6. In accordance, participants were divided in two groups, “under median value (≤50%)” (*n* = 50) and “above median value (>50%)” (*n* = 50). Thus, the “under median value (≤50%)” group was made up of participants with values ≤ 2.6 kg CO_2_/day and the “above median value (>50%)” group comprised participants with values >2.6 kg CO_2_/day. 

The experimental protocol adhered to the ethical standards outlined in the Declaration of Helsinki. Approval for all procedures was obtained from the Ethics Committee of Research of Balearic Islands (CEIC-IB/2251/14PI; approved on 26 February 2020). Additionally, participants were fully informed about this study’s objectives and implications, and each participant provided written informed consent.

### 2.2. Anthropometric Measurements

Registered dietitians measured all the anthropometric parameters. Body weight (kg) was assessed using a segmental body composition analyzer following the manufacturer’s guidelines (Tanita BC-418, Tanita, Tokyo, Japan). The measurements were taken with participants barefoot and wearing light clothing, with an adjustment of 0.6 kg subtracted. Height (cm) was determined using a mobile anthropometer (Seca 213, SECA Deutschland, Hamburg, Germany), with the patient’s head maintained in the Frankfort horizontal plane position. Body mass index (BMI) was determined according to weight (kg)/height (m^2^). Dividing waist (cm) with height (cm), the waist-to-height ratio (WHtR) was calculated. Abdominal obesity (cm) was determined in duplicate, using an anthropometric tape halfway between the last rib and the iliac crest. Blood pressure was determined in a seated position, in triplicate, with a validated semiautomatic oscillometer (Omron HEM, 705CP, Hoofdrop, The Netherlands).

### 2.3. Blood Collection and Analysis

Blood samples were obtained under overnight fasting conditions from the antecubital vein using appropriate vacutainers containing ethylenediaminetetraacetic acid (EDTA) as an anticoagulant. Biochemical parameters: glucose, glycosylated hemoglobin (HbA1c), triglycerides (TG), total cholesterol, high-density lipoprotein cholesterol (HDL-cholesterol), low-density lipoprotein cholesterol (LDL-cholesterol), aspartate aminotransferase (AST), alanine aminotransferase (ALT), and γ-glutamyl transferase (GGT) were measured using standardized clinical procedures. 

Blood cell counts and hematological parameters were assessed in whole blood using an automated flow cytometer analyzer (Technion H2, Bayer, VCS system, Frankfurt, Germany). Urine samples were collected after a 12-h overnight fasting period, specifically from the first urine of the day, and placed in a sterilized container. Uric acid, creatinine in serum, and albumin and creatinine in urine were also determined. Estimated glomerular filtration (eGFR) was calculated using the Chronic Kidney Disease Epidemiology Collaboration (CKD-EPI) equation [22].

### 2.4. Blood Samples Processing 

Plasma samples were gathered after centrifugation at 1700× *g* for 15 min at 4 °C of whole fresh blood. The peripheral blood mononuclear cell (PBMC) fraction was extracted from freshly collected whole blood and isolated according to the provided protocol for white blood cell separation [23], using the reagent Ficoll-Paque PLUS (GE Healthcare Bio-Science, AB, Uppsala, Sweden) [24]. 

### 2.5. Fatty Liver Index

The fatty liver index (FLI) was computed using the formula below [25]:

$$Fatty\;Liver\;Index\;\left(FLI\right)=\frac{e^{y}}{\left(1+e^{y}\right)}\times 100$$

where:

$$y=0.953\times \ln\left(TG\right)+0.139\times BMI+0.718\times \ln\left(GGT\right)+0.053\times WC-15.745$$

Abbreviations and units: TG—triglycerides (mg/dL); BMI—body mass index (kg/m^2^); GGT—γ-glutamyl transferase (U/L); WC—waist circumference (cm).

### 2.6. Dietary Inflammatory Index

The diet inflammatory index (DII) is used to evaluate the inflammatory potential of a diet [26]. This score derives from incorporating the effect of 45 food items on six inflammatory biomarkers (IL-10, IL-1β, IL-6, IL-4, TNFα, and highly sensitive C-reactive protein (CRP)) [26]. The effect of the diet was assessed as anti-inflammatory with a negative food score, as proinflammatory with a positive food score, and as no significant changes in inflammatory biomarkers with a food score equal to 0. This index was determined using dietary intake derived from the validated FFQ, previously described [17].

### 2.7. Assessment of Mediterranean Diet Adherence

Adherence to the Mediterranean diet (MedDiet) was calculated through a validated questionnaire of 17 items [27]. The overall score ranged from 0 to 17, with a score of 0 signifying noncompliance and a score of 17 indicating the highest level of adherence.

### 2.8. Enzymatic Determinations

Following the spectrophotometric method of Aebi, catalase (CAT) activity was determined based on the decomposition of H_2_O_2_ [28]. Superoxide dismutase (SOD) activity was assessed using a modified version of the Flohe and Otting method, which relies on inhibiting the reduction of cytochrome C by superoxide anion [29]. The measurement of both antioxidant enzymes in plasma samples was conducted using a Shimadzu UV-2100 spectrophotometer (Shimadzu Corporation, Kyoto, Japan) at a temperature of 37 °C.

### 2.9. Malondialdehyde Assay

Malondialdehyde (MDA) was determined in plasma and urine samples using a colorimetric assay kit (Sigma-Aldrich Merck^®^, St. Louis, MO, USA). The reaction of MDA with a chromogenic reagent is accompanied by the formation of a stable chromophore. Standards and samples were added in tubes with *n*-methyl-2-phenylindole in acetonitrile: methanol (3:1) mixture. After that, 12N HCl was added and thereafter a sample was incubated for 1 h at 45%. Finally, the absorbance was assessed at 586 nm, and the concentration of MDA was computed utilizing a standard curve with known concentrations ranging from 0 to 20 nM.

### 2.10. Polyphenols Determination

The concentration of polyphenols was measured in urine-deproteinized and plasma samples with cold acetone (1:1.2), following the Folin–Ciocalteu method [30], using L-tyrosine as the standard.

### 2.11. ROS Production in Neutrophils and PBMCs 

In neutrophils and PBMCs, ROS production was determined after being activated with zymosan A (ZYM) (1 mg/mL PBS) from *Saccharomyces cerevisiae* (Sigma-Aldrich) and lipopolysaccharide (LPS) (100 µg/mL phosphate-buffered saline (PBS)), following the previously described protocol [31].

### 2.12. Immunoassay Kits

Plasma levels of monocyte chemotactic protein 1 (MCP-1) were assessed using specific ELISA kits (RayBiotech^®^, Parkway Lane, Suite, Norcross, GA, USA) as per the provided manufacturer’s guidelines. The intraassay coefficient of variation for MCP-1 was determined as <10%, and the interassay coefficient of variation was <12%. Tumor necrosis factor α (TNFα) levels in plasma were quantified utilizing an ELISA kit (Diaclone, Besancon CEDEX, France) with intraassay and interassay coefficients of variation of 3.2% and 10.9%, respectively. Interleukin 15 (IL-15), resistin, ghrelin, and leptin were measured in plasma using Human Custom ProcartaPlexTM (Invitrogen by Thermo Fisher Scientific, Bender MedSystems GmbH, Vienna, Austria) following the specified instructions. All immunoassays were conducted on a microplate reader set at 450 nm (Epoch, Bioteck Instruments, Bad Friedrichshall, Germany).

### 2.13. Statistics

The statistical analysis was conducted using the Statistical Package for Social Sciences version 28.0 (SPSS v.28 for Windows, IBM Software Group, Chicago, IL, USA). Descriptive statistics, including the mean and standard deviation (SD), were employed for each variable. Participants were categorized into two groups based on the daily emissions of CO_2_ in kilograms (≤2.6 kg CO_2_/day, *n* = 50; >2.6 kg CO_2_/day, *n* = 50). Statistical differences were assessed using Student’s *t*-test for normally distributed variables and the Mann–Whitney U test for nonnormally distributed variables. Results were deemed statistically significant if the *p*-value was <0.05. The odds ratio (OR) and 95% confidence interval (CI) were computed to explore the association between oxidative stress, inflammatory biomarkers, and dietary CO_2_ emissions.

## 3. Results

The study subjects consisted of adults aged 55 to 75 years, sampled in the Balearic Islands (Spain). All the participants met the MetS criteria and, as a whole, presented values of the five criteria that define the MetS outside the reference values for the healthy population (Table 1). In addition, subjects had a BMI greater than 30, indicative of a state of obesity. No significant differences were detected between the two groups of participants, classified according to dietary CO_2_ emissions in anthropometric parameters and general biochemistry. Higher DII values were observed in participants with higher kg CO_2_ emissions, while adherence to MedDiet values were lower.

When analyzing the results of hematological and urine parameters of the participants according to the dietary kg CO_2_ emissions (Table 2), no significant differences were detected in any of the variables analyzed. Specifically, the number of circulating cells and urinary markers of renal function were similar in both groups.

The results of oxidative damage and oxidative stress biomarkers measured in blood and urine stratified by kg of dietary CO_2_ emissions are shown in Table 3. Although no significant differences were detected between the activities of plasma antioxidant enzymes, higher significant levels of MDA in urine and plasma were found in the group with higher dietary CO_2_ emissions compared to the group with reduced dietary emissions. Moreover, a significantly lower concentration of polyphenols was evidenced in the group with higher dietary CO_2_ emissions, but no significant differences were observed in urine polyphenol levels. Moreover, ROS production determined in PBMCs stimulated with zymosan and LPS presented significantly greater levels in the group with greater dietary CO_2_ emissions. Similar results were obtained in neutrophils stimulated with LPS, whereas no significant differences were reported when stimulated with zymosan. 

**Figure 1:**
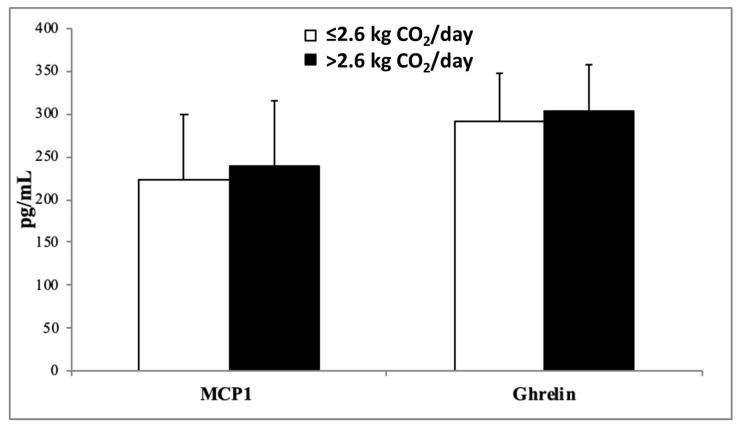
Monocyte chemoattractant protein 1 (MCP-1) and Ghrelin levels in plasma classified according to dietary kg CO_2_ emissions. Results are shown as mean (SD). Statistical analysis: Student’s *t*-test for impaired data or Mann–Whitney U test.

**Figure 2:**
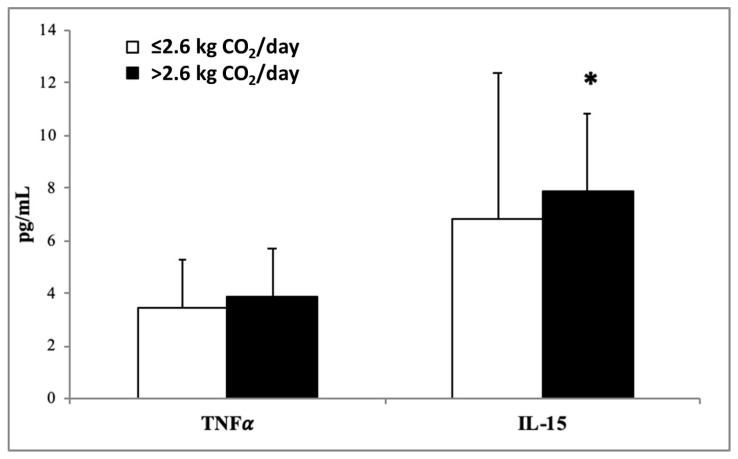
Tumor necrosis factor α (TNFα) and interleukin 15 (IL-15) levels in plasma classified according to dietary kg CO_2_ emissions. Results are shown as mean (SD). Statistical analysis: Student’s *t*-test for impaired data or Mann–Whitney U test. * *p* < 0.05.

**Figure 3:**
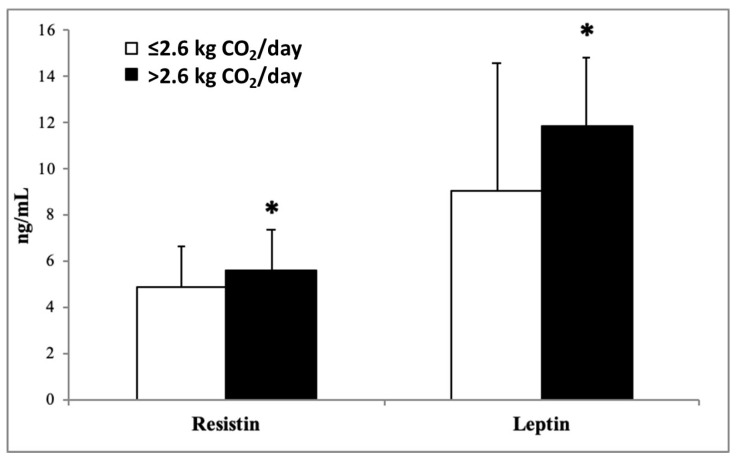
Resistin and leptin levels in plasma classified according to dietary kg CO_2_ emissions. Results are shown as mean (SD). Statistical analysis: Student’s *t*-test for impaired data or Mann–Whitney U test. * *p* < 0.05.

The plasma levels of inflammatory biomarkers, MCP-1, Ghrelin (Figure 1), TNFα, IL-15 (Figure 2), resistin, and leptin (Figure 3) in participants stratified by dietary CO_2_ emissions are shown below. All inflammatory parameters showed a tendency to be higher in the group that produced more CO_2_ emissions, suggesting a greater proinflammatory state, although the differences were only statistically significant for IL-15, resistin, and leptin.

Adjusted ORs were determined to assess the possible relationship between oxidative stress and inflammatory biomarkers and dietary CO_2_ emissions (Table 4), considering the low CO_2_ emissions group as reference. OR analysis, adjusted for age and sex, revealed that higher emission of dietary CO_2_ was a potential factor affecting ROS production in PBMCs and neutrophils stimulated by zymosan, IL-15, and resistin levels, contributing to an increase in the prooxidant and inflammatory state. 

## 4. Discussion

The key discoveries of this research indicate a correlation between dietary carbon dioxide emissions and oxidative stress as well as proinflammatory biomarkers. The results revealed that participants with high DII were those with high dietary CO_2_ emissions. Thus, these results show that diets with a more proinflammatory profile become unhealthier and less sustainable. A proinflammatory diet is characterized by ultra-processed food and beverage products (such as commercial pastries, sauces, and preprepared dishes manufactured with additives) and low intake of fruit and vegetables. This diet is increasing due to globalization, especially in countries growing richer [32]. The adherence to the MedDiet was inversely associated with CO_2_ emissions. Previously, it was reported that better adherence to the MedDiet is an ecofriendly option because of its relation to lower land use, water and energy consumption, and GHG emissions [33]. 

Regarding oxidative stress and oxidative damage markers, this study revealed that participants with high dietary CO_2_ emissions showed a significant increase in MDA levels in both plasma and urine samples. MDA is used as a marker of oxidative damage, as it is generated as a byproduct of unsaturated fatty acid peroxidation [34,35]. In fact, increased ROS production is considered the primary source of MDA in inflammatory disorders [36]. Moreover, it has been evidenced that high food quality characterized by high fruit and vegetable intake is associated with reduced levels of inflammation and oxidative stress [37,38]. These findings are in accordance with the current results, since an unhealthy diet characterized by higher CO_2_ emissions presented significantly higher values of MDA levels. Another association with quality of diet is plasma and urine polyphenol levels. Polyphenols are defined as a complex group of compounds naturally occurring in plant foods with diverse biological functions including antioxidant and anti-inflammatory. Polyphenols could improve cardiovascular health owing to their high antioxidant potential [39]. Polyphenols contribute to protecting against adipose tissue expansion during the process of weight gain because they can modulate gene expression [40]. The current results revealed that dietary CO_2_ emissions are significantly associated with low polyphenol levels in plasma and urine and, consequently, these results show that a diet with high CO_2_ emissions is a poor-quality diet. The implications of polyphenol intake on health are evident in the relationship between increased consumption of polyphenols and high excretion in urine with better endothelial function and a reduction in blood pressure [30].

The current findings also evidenced that participants with high dietary CO_2_ emission did not show significantly high activities of CAT and SOD (antioxidant enzymes), evidencing an imbalance between the antioxidant defense mechanism and production of ROS, thus increasing the risk of oxidative stress creation. A previous study found a correlation between a high concentration of circulating triglycerides with enhanced ROS production by neutrophils in obese subjects [41]. A rise in ROS production and the activities of plasma antioxidant enzymes were observed in overweight and obese participants, compared to normal-weight subjects [42,43], to protect against the oxidative stress produced by excess high-fat and/or high-carbohydrate diets [44]. During the current study, all participants had MetS and, consequently, a common problem; however, it can be observed that the less healthy and more prooxidative and proinflammatory diet produced more CO_2_ but was not related to an antioxidant response.

The current study revealed that inflammatory mediators in plasma, such as IL-15, resistin, and leptin, showed higher significant levels in subjects with high dietary CO_2_ emissions than in patients with low dietary CO_2_ emissions. IL-15 is defined as a proinflammatory cytokine included in the development of low-grade chronic inflammation present in MetS related to obesity [45]. High inflammatory cytokine levels are related to noncommunicable diseases and can be induced by a macronutrient profile intake [46]. While the caloric content of macronutrients (carbohydrates, proteins, and lipids) is important, their balance and quality should also be considered for a healthy diet [47]. Western diets contain excessively refined sugar, excess trans fatty acids and saturated fats, and an unhealthy balance of omega-6 and omega-3 fatty acids [48]. A positive correlation was observed between lipid hydroperoxide (LOOH) and levels of IL-15, TNFα, insulin, total cholesterol, LDL, and triglycerides in normal-weight obese syndrome patients [49]. The current results are in accordance with a previous study that showed an association between cytokines and the metabolic score for insulin resistance, with BMI as a significant mediator [50].

Resistin is defined as a hormone secreted from adipose tissue that is related to the development of type 2 diabetes mellitus, since it appears a connecting link between visceral obesity and diabetes [51,52]. Human resistin deals with various physiological functions and is implicated in the secretion of immune effectors such as TNF-α, IL-1β, IL-6, IL-8, and IL-12, which stimulate the inflammatory response [53,54]. Furthermore, resistin has been proposed as a biological marker due to its relationships to chronic diseases, such as cardiovascular diseases and cancers [55]. The current results revealed a direct association between levels of resistin in plasma and dietary CO_2_ emissions.

Leptin is a hormone derived from adipocytes and is the major regulator for food intake and energy homeostasis [56]. A previous study observed that diets rich in sugar can lead to abnormalities associated with increased coronary heart disease, including leptin resistance, high levels of glucose, insulin, and uric acid [57]. Therefore, in the current study, the higher levels of leptin in participants with high dietary CO_2_ emissions reinforce the idea that these participants had an unhealthier diet, with concentrated sugars such as high-fructose and sucrose in the form of ultra-processed foods [57]. Also, a previous study demonstrated that high levels of inflammatory plasma markers, such as IL-6, IL-1β, IL-15, TNF-α, and resistin, in MetS patients are related to low adherence to MedDiet [16].

Lastly, the present findings from the odds ratio test show that the plasma levels of IL-15 and resistin, ROS production stimulated by zymosan in PBMCs, and neutrophils may be good biomarkers of dietary CO_2_ emissions.

### Strengths and Limitations

The primary highlight of this study lies in its groundbreaking revelation that dietary CO_2_ emissions are, for the first time, correlated with oxidative stress and a proinflammatory state among individuals sharing similar characteristics. This connection is attributed to the impact of globalization and the abandonment of traditional, healthy dietary patterns. However, a noteworthy drawback of this research is the relatively small sample size. Despite this limitation, it is crucial to note that the sample size proved sufficient to detect discernible variations in inflammatory and oxidative stress biomarkers between the high CO_2_ emissions group and the low CO_2_ emissions group.

## 5. Conclusions

Participants with high dietary CO_2_ emissions showed worse quality diet and oxidative and inflammatory status than patients with low CO_2_ emissions. Specifically, high CO_2_ emissions were related to high DII, MDA, ROS production, IL-15, resistin, and leptin levels, and decreased adherence to MedDiet and polyphenol levels in plasma. Consequently, it is necessary to promote healthy eating to reduce sociosanitary problems and as a way of living and respecting the environment.

## Figures and Tables

**Table 1:** Anthropometric and biochemical parameters stratified by dietary kg CO_2_ emissions.

	≤2.6 kg CO_2_/day *n* = 50	>2.6 kg CO_2_/day *n* = 50	*p*-Value
	Mean (SD)	Mean (SD)	
Weight (kg)	87.4 (13.9)	87.8 (13.5)	0.794
Height (cm)	162.6 (9.0)	163.0 (9.6)	0.720
BMI (kg/m^2^)	32.9 (3.6)	32.9 (3.6)	0.936
WHtR	0.687 (0.6)	0.681 (0.5)	0.368
Abdominal obesity (cm)	111.1 (10.2)	111.4 (10.1)	0.786
Systolic blood pressure (mmHg)	138.6 (15.9)	138.9 (18.1)	0.907
Diastolic blood pressure (mmHg)	80.3 (9.9)	80.6 (9.5)	0.908
Glucose (mg/dL)	118.3 (36.2)	117.8 (35.7)	0.896
HbA1c (%)	6.19 (1.1)	6.25 (1.21)	0.689
Triglycerides (mg/dL)	148.5 (70.4)	154.0 (73.6)	0.531
HDL-cholesterol (mg/dL)	44.2 (10.9)	44.2 (9.9)	0.971
LDL-cholesterol (mg/dL)	117.6 (34.8)	120.9 (35.0)	0.445
Total cholesterol (mg/dL)	184.1 (38.1)	186.0 (38.8)	0.692
AST (U/L)	20.3 (5.1)	20.1 (6.4)	0.798
ALT (U/L)	22.7 (11.8)	22.1 (9.8)	0.652
GGT (U/L)	31.9 (22.9)	33.4 (24.3)	0.611
FLI	79.0 (18.1)	80.8 (16.3)	0.389
DII	−0.17 (2.2)	0.82 (2.0)	<0.001
ADM	7.92 (2.6)	7.07 (2.3)	0.004

Abbreviations: ADM: adherence to Mediterranean diet; BMI: body mass index; DII: dietary inflammatory index; FLI: fatty liver index; SD: standard deviation; WHtR: waist-to-height ratio. Results are expressed as mean (SD). Statistical analysis: Student’s *t*-test for impaired data. Kg of CO_2_ per consumed food = Kg of each consumed food from FFQ*Kg CO_2_ emitted per kg of each food (EU database 2016).

**Table 2:** Hematological and urine parameters stratified by dietary kg CO_2_ emissions.

	≤2.6 kg CO_2_/day *n* = 50	>2.6 kg CO_2_/day *n* = 50	*p*-Value
	Mean (SD)	Mean (SD)	
Hematocrit (%)	42.9 (4.1)	43.1 (3.9)	0.688
Erythrocytes (10^6^/mm^3^)	4.79 (0.5)	4.78 (0.5)	0.962
Leukocytes (10^3^/mm^3^)	7.31 (1.7)	7.40 (1.8)	0.664
Neutrophils (10^3^/mm^3^)	4.34 (2.8)	4.41 (4.1)	0.870
Lymphocytes (10^3^/mm^3^)	2.59 (2.3)	2.42 (0.8)	0.411
Monocytes (10^3^/mm^3^)	0.68 (0.6)	0.73 (0.8)	0.574
Eosinophils (10^3^/mm^3^)	0.24 (0.5)	0.29 (0.4)	0.340
Basophils (10^3^/mm^3^)	0.05 (0.1)	0.09 (0.4)	0.277
Hemoglobin (g/dL)	14.5 (1.4)	14.5 (1.5)	0.913
Medium corpuscular volume	89.9 (5.4)	90.4 (4.6)	0.476
Platelets	218.0 (57.2)	224.6 (56.6)	0.344
Uric acid (mg/dL)	6.33 (1.4)	6.14 (1.3)	0.269
Creatinine in serum (mg/dL)	0.83 (0.2)	0.84 (0.2)	0.648
Urine albumin (mg/dL)	19.4 (23.1)	21.4 (27.6)	0.594
Urine creatinine (mg/dL)	95.2 (53.9)	102.8 (50.7)	0.242
Albumin/creatinine ratio in mg/g creatinine (uACR)	22.4 (29.7)	24.3 (32.2)	0.315
eGFR (mL/min per 1.73 m^2^)	81.3 (19.1)	84.0 (17.2)	0.234

Abbreviations: eGFR: estimated glomerular filtration rate; SD: standard deviation; uACR: urine albumin–creatinine ratio (uACR). Results are expressed as mean (SD). Statistical analysis: Student’s *t*-test for impaired data or Mann–Whitney U test. Kg of CO_2_ per consumed food = Kg of each consumed food from FFQ*Kg CO_2_ emitted per kg of each food (EU database 2016).

**Table 3:** Oxidative stress biomarkers stratified by dietary kg CO_2_ emissions.

	≤2.6 kg CO_2_/day *n* = 50	>2.6 kg CO_2_/day *n* = 50	*p*-Value
	Mean (SD)	Mean (SD)	
Plasma enzymes			
CAT (kat/L blood)	49.6 (23.6)	53.4 (24.3)	0.336
SOD (pkat/L blood)	153.9 (64.3)	164.7 (88.8)	0.421
Oxidative damage			
MDA plasma (nM)	1.08 (0.55)	1.36 (0.58)	0.003
MDA urine/creatinine (mM/mM)	80.9 (45.7)	115.2 (98.9)	0.005
Polyphenols plasma (mg/mL)	0.06 (0.02)	0.05 (0.01)	0.029
Polyphenols urine/creatinine (g/L/mM)	15.6 (12.6)	12.6 (8.2)	0.079
ROS production			
PBMCs Zym (RLU/min·10^3^ cells)	3024 (1519)	3737 (2081)	0.002
PBMCs LPS (RLU/min·10^3^ cells)	1190 (670)	1444 (943)	0.010
Neutrophils Zym (RLU/min·10^3^ cells)	10,538 (5679)	11,831 (5818)	0.056
Neutrophils LPS (RLU/min·10^3^ cells)	2994 (1414)	3612 (1912)	0.016

Abbreviations: CAT, Catalase; MDA; Malondialdehyde; Neutrophils LPS, Neutrophils stimulated with lipopolysaccharide; Neutrophils Zym, Neutrophils stimulated with Zymosan; PBMCs LPS, Peripheral blood mononuclear cell stimulated with lipopolysaccharide; PBMCs Zym, Peripheral blood mononuclear cell stimulated with Zymosan; SD, Standard Deviation; SOD, Superoxide dismutase. Results are expressed as mean (SD). Statistical analysis: Student’s *t*-test for impaired data or Mann Withney’s U-test. Kg of CO_2_ per consumed food = Kg of each consumed food from FFQ*Kg CO_2_ emitted per kg of each food (EU data base 2016).

**Table 4:** Association (odds ratio and 95% confidence interval) between the oxidative stress and inflammation biomarkers (dependent variables) and dietary kg CO_2_ emissions (independent variables).

Variables	≤2.6 kg CO_2_/day *n* = 50	>2.6 kg CO_2_/day *n* = 50	*p*-Value
	OR (95% CI)	OR (95% CI)	
CAT plasma	1.00 (ref.)	1.18 (0.606–2.296)	0.628
SOD plasma	1.00 (ref.)	0.70 (0.338–1.464)	0.348
MDA plasma	1.00 (ref.)	1.45 (0.761–2.774)	0.258
MDA urine/creatinine	1.00 (ref.)	0.96 (0.342–2.657)	0.927
Polyphenols plasma	1.00 (ref.)	1.15 (0.681–1.943)	0.601
Polyphenols urine/creatinine	1.00 (ref.)	0.92 (0.429–1.964)	0.826
ROS PBMCs Zym	1.00 (ref.)	3.75 (2.166–6.491)	<0.001
ROS PBMCs LPS	1.00 (ref.)	1.27 (0.783–2.071)	0.330
ROS neutrophils Zym	1.00 (ref.)	1.75 (1.093–2.809)	0.020
ROS neutrophils LPS	1.00 (ref.)	1.09 (0.664–1.782)	0.738
MCP-1	1.00 (ref.)	1.13 (0.604–2.082)	0.716
TNFα	1.00 (ref.)	1.84 (0.828–4.089)	0.134
IL-15	1.00 (ref.)	2.34 (0.999–5.482)	0.050
Resistin	1.00 (ref.)	3.06 (1.392–6.706)	0.005
Ghrelin	1.00 (ref.)	1.66 (0.779–3.528)	0.190
Leptin	1.00 (ref.)	1.86 (0.764–4.522)	0.172

Abbreviations: CAT: catalase; IL-15: interleukin 15; MDA: malondialdehyde; MCP-1: monocyte chemoattractant protein 1; neutrophils Zym: neutrophils stimulated with zymosan; neutrophils LPS: neutrophils stimulated with lipopolysaccharide; OR adjusted: odds ratio after adjustments for sex and age; PBMCs Zym: peripheral blood mononuclear cell stimulated with zymosan; PBMCs LPS: peripheral blood mononuclear cells stimulated with lipopolysaccharide; SOD: superoxide dismutase; TNFα: tumor necrosis factor α.

## Data Availability

There are restrictions on the availability of data for this trial due to the signed consent agreements around data sharing. Researchers wishing to access the trial data used in this study can make a request to the corresponding author: pep.tur@uib.es.
